# Measuring hospital spatial accessibility using the enhanced two-step floating catchment area method to assess the impact of spatial accessibility to hospital and non-hospital care on the length of hospital stay

**DOI:** 10.1186/s12913-021-07046-3

**Published:** 2021-10-11

**Authors:** Fei Gao, Matthieu Jaffrelot, Séverine Deguen

**Affiliations:** 1 Department of Quantitative Methods for Public Health, EHESP School of Public Health, Rennes, Avenue du Professeur Léon Bernard, 35043 Rennes, France; 2 L’équipe REPERES, Recherche en Pharmaco-épidémiologie et recours aux soins, UPRES EA-7449, Rennes, France; 3 Univ Rennes, Ensai, F-35000 Rennes, France; 4 IPLESP, Department of Social Epidemiology, INSERM, Sorbonne Université, Institut Pierre Louis d’Épidémiologie et de Santé Publique, F75012 Paris, France

**Keywords:** Potential accessibility, Hospital care, Non-hospital care, Length of stay, E2SFCA

## Abstract

**Background:**

Optimal healthcare access improves the health status and decreases health inequalities. Many studies demonstrated the importance of spatial access to healthcare facilities in health outcomes, particularly using the enhanced two-step floating catchment area (E2SFCA) method. The study objectives were to build a hospital facility access indicator at a fine geographic scale, and then to assess the impact of spatial accessibility to inpatient hospital and non-hospital care services on the length of hospital stay (LOS).

**Methods:**

Data concerning older adults (≥75 years) living in the Nord administrative region of France were used. Hospital spatial accessibility was computed with the E2SFCA method, and the LOS score was calculated from the French national hospital activity and patient discharge database. The relationship between LOS and spatial accessibility to inpatient hospital care and to three non-hospital care types (general practitioners, physiotherapists, and home-visiting nurses) was analyzed with linear regression models.

**Results:**

The mean number (standard deviation) of beds per 10,000 inhabitants was 19.0 (10.69) in Medical, Surgical and Obstetrics (MCO) facilities and 5.58 (2.19) in Postoperative and Rehabilitation Care (SSR) facilities, highlighting important variations within the region. Accessibility to hospital services was higher in large urban areas, despite the dense population and higher demand. In 2014, the mean LOS scores were 0.26 for MCO and 0.85 for SSR, but their geographical repartition was non-homogeneous. The linear regression analysis revealed a strong negative and significant association between LOS and non-hospital care accessibility.

**Conclusions:**

This is the first study to measure spatial accessibility to inpatient hospital care in France using the E2SFCA method, and to investigate the relationship between healthcare utilization (LOS score) and spatial accessibility to inpatient hospital care facilities and three types of non-hospital care services. Our findings might help to make decisions about deploying additional beds and to identify the best locations for non-hospital care services. They might also contribute to improve access, and to ensure the best coordination and sustainability of inpatient and outpatient services, in order to better cover the population’s healthcare needs. International studies using multiple consensual indicators of healthcare outcomes and accessibility and sophisticated modeling methods are needed.

## Background

### Healthcare access definition

Access to healthcare is widely recognized as the foundation of any high-performing healthcare system. Ensuring a high degree of healthcare access improves people’s health status and decreases health inequalities. Equal accessibility to healthcare facilities for everyone is an essential goal for international political organizations and national governments [1–5].

Access to healthcare can be defined in different ways. One of the most common definitions was developed by Andersen, and characterizes this access along two dimensions: potential (or spatial) and realized access [6, 7]. Spatial access describes the offer of healthcare facilities in a given area, and the modality/time potentially needed to go to one of such facilities/services from a given position or by a person [8–12]. As spatial access varies across space and time, it is affected by location relationships and travel impedance. On the other hand, realized access describes the actual use of the available healthcare services/facilities [13] and the real interactions with the healthcare system [14]. Penchansky and Thomas [15] grouped barriers that limit the passage from potential to realized access in five dimensions: availability, accessibility, affordability, acceptability, and accommodation. These authors showed that potential and realized access are two separated, but closely related notions. Indeed, potential access could have a great impact on healthcare utilization behaviors. On the other hand, realized access could influence the spatial organization of healthcare services. Many studies have assessed the potential access to healthcare services and care utilization [16, 17]. Conversely, until recently, research on how potential access may influence utilization patterns was limited [18–20]. Yet, the links between potential and realized access must be investigated because they can bring crucial insights that can be used to better understand the demands by healthcare users and to inform healthcare providers and political actors.

### Assessing spatial accessibility to non-hospital care and to inpatient hospital care

The assessment of access to healthcare services should take into account the care provided by various facility types. In many countries, the healthcare system is based on a combined architecture: non-hospital care, based on general practitioners who can address patients to other healthcare professionals (e.g. nurses, physiotherapists, specialists doctors), and hospital care [21]. Non-hospital care represents the most significant primary care contributor in many countries, including developing countries [22, 23]. Indeed, it ensures an effective and generally faster service that covers the large majority of personal healthcare needs [24], and acts as the principal point of continuing care for patients [25]. Nevertheless, hospitals remain key healthcare actors. Inpatient care provided by hospitals represents a major part of healthcare and medical good consumption, especially in Europe. In 2017, nine of the 10 countries with the highest hospital discharge rates worldwide were European Union member states [26]. Coordination and organization between inpatient hospital care and primary care are critical for a successful healthcare system, particularly during a pandemic when the services offered by hospital facilities can become saturated. In these situations, non-hospital resources (i.e. the most significant primary care contributor) could anticipate and limit the number of hospitalizations and the consumption of hospital-linked resources [27, 28]. A consolidated spatial organization of non-hospital medical services in the territory can complement hospital services and increase healthcare efficiency [29].

One of the most widely used approaches to assess spatial accessibility is the gravity-based enhanced two-step floating catchment area (E2SFCA) method. Two different spatial indicators have been constructed in France: i) the localized potential accessibility score (Accessibilité potentielle localisée: APL) developed by the French Institute for Research and Information in Health Economics at the municipality level in 2011 [30], and at the census block level for the Greater Paris area in 2019 [31]; and the index of spatial accessibility (ISA) at the census block level, implemented by Gao and al. in 2016 [32]. However, these previous studies focused only on non-hospital care services. So far, no French indicator based on the E2SFCA method has measured the access to hospital care at a very fine geographic scale.

### Assessing interactions

It is essential to assess accessibility to non-hospital care and inpatient hospital care, and also to examine their interactions and with healthcare utilization. It has been hypothesized that in the presence of geographic barriers that limit access to primary care, hospital services might be used more frequently [33–35], for instance by people living in medically underserved areas [34, 36, 37]. Moreover, the capacity of primary healthcare services to take care of discharged patients has a significant effect on hospitalization length [21, 38, 39], particularly for elderly people [40, 41]. This suggests that the length of hospital stay (LOS), one of the classical indicators of healthcare utilization, is influenced by the primary care offer. This indicator may help to explore the interactions between non-hospital care and inpatient hospital care, and between healthcare accessibility and utilization.

In this context, the main aim of this study was to analyze the relationship between healthcare spatial accessibility and health service utilization following three main complementary steps: 1) to build an inpatient hospital care access indicator at a fine geographic scale; 2) to measure health service utilization using the LOS; and 3) to explore the interaction between access and utilization by investigating the impact of spatial accessibility to inpatient hospital care and non-hospital healthcare services on the LOS. To this purpose, data on the older adults (≥75-year of age) living in the Nord administrative region of France were used. Indeed, older adults represent a growing proportion of the total population that is expected to double by 2050 [42]. In France, the proportion of ≥75-year-old adults was 9.7% in 2020 and is expected to reach 16% by 2050 [43]. As this population present many age-related diseases (e.g. chronic diseases), different healthcare resources are involved in their management: hospital facilities and primary care professionals (e.g. general practitioners, physiotherapists, and home-visiting nurses). Additionally, as their recovery period after a hospital stay is often longer, their LOS could be more influenced by the ability of the primary healthcare services to follow them.

## Methods

### Study setting and population

The Nord administrative region has a surface area of 5743 km^2^ and a population density of 456 inhabitants per km^2^. This region was selected due to the availability of several metrics of non-hospital care accessibility [30–32], and because edge effects influence only slightly accessibility to hospital services/facilities in this area [44]. The study concerned only ≥75-year-old adults.

### Data sources and statistical unit

Various data sources were combined for the present study:

1. The accessibility to non-hospital care was described using the APL database [45]. Nation-wide APL indices have been computed using the E2SFCA method for eight types of self-employed practitioners working in primary care: general practitioners, physiotherapists, home-visiting nurses, gynecologists, dental surgeons, midwives, pediatricians, and ophthalmologists. General practitioners, physiotherapists, and home-visiting nurses are significantly implicated in the management of ≥75-year-old people, and thus might influence the need/duration of inpatient hospital care. Therefore, their APL indices were selected for this analysis;
2. The number of available beds in each hospital was extracted from the Annual Statistical Survey of Healthcare Facilities 2014 database (Statistique Annuelle des Établissements) [46]. Facilities were classified in two categories: Medical, Surgical and Obstetrics facilities (Médecine, Chirurgie, Obstétrique: MCO) and Postoperative and Rehabilitation Care facilities (Soins de Suite et de Réadaptation: SSR);
3. The postal address of each hospital was obtained from the National File on Health and Social Institutions (Fichier national des établissements sanitaires et sociaux: FINESS) [47], and then converted into latitude and longitude using the French National Address Database [48];
4. Data for 2014 from the French national discharge database [49–52] were used to calculate the LOS for MCO and SSR facilities;
5. The number of ≥75-year-old adults in the region under study was extracted from the 2016 French national census [53].

The statistical unit was the French Geographic Code (FGC). This metrics is used in the French national discharge database, and corresponds to the postal code of the city of residence.

### Methodology

First, the E2SFCA method was implemented to compute the hospital spatial accessibility by combining geographical, supply and demand factors. Second, the LOS indicator was estimated for each FGC unit. Third, linear regression models were used to analyze the relationship between LOS and spatial accessibility to inpatient hospital care and also to the three types of non-hospital care services (general practitioners, physiotherapists, and home-visiting nurses). The final variables included in the model for additional analyses and the categories of facilities for which they are available are shown in Table 1.

**Table 1:** Description of the dependent and independent variables used in this study

VARIABLES	DESCRIPTION	Category of facilities
Dependent variables		
LOS_MCO/ LOS_SSR	The mean hospital stay length in MCO or SSR of elderly people (≥75 years of age) relative to the total ≥ 75-year-old population	Hospital
Independent variables		
ISA_MCO/ ISA_SSR	Index of spatial accessibility to MCO and SSR facilities	Hospital
APL_GPs	Localized Potential Accessibility to general practitioners	Non-hospital
APL_Nurses	Localized Potential Accessibility to home-visiting nurses	Non-hospital
APL_Physiotherapists	Localized Potential Accessibility to physiotherapists	Non-hospital
Composite_APL	Built from APL_GPs, APL_Nurses and APL_Physiotherapists using principal component analysis	Non-hospital

*MCO* Medical, Surgical and Obstetrics, *SSR* Postoperative and Rehabilitation Care

#### Assessing hospital care spatial accessibility using the E2SFCA method

The hospital facility access indicator was built in two steps using the E2SFCA method [54, 55].

In step 1, for each hospital center *j* with a MCO or SSR facility, the number of beds in the MCO or SSR facility *S*_*j*_ was counted, and the population living in the FGC *k* and located within a threshold drive time *d*_*max*_ from the hospital center *j* (i.e. catchment area *j*) was estimated. Then, the bed-to-population ratio *R*_*j*_ within the catchment area *j* was determined with (Eq. 1):

1 $$ {R}_j=\frac{S_j}{\sum \limits_{k\in \left\{{d}_{kj}\le {d}_{max}\right\}}{P}_k\ast w\left({d}_{kj}\right)} $$

where *P*_*k*_ is the patient population in the FGC *k* the centroid of which falls within the catchment area *j* (i.e. *d*_*kj*_ *< d*_*max*_), *S*_*j*_ is the number of beds available in the hospital center *j*, *d*_*kj*_ is the driving time between the FGC *k* and the hospital center *j*, and *w()* is a weighted decay function that depends on the driving time *d*_*kj*_.

In step 2, for each population location *i*, all MCO or SSR facility locations *j* that were within the threshold driving time *d*_*max*_ from location *i* (i.e. the catchment area *i*) were estimated, and all *R*_*k*_ for the catchment area were summed to calculate the Index of Spatial Accessibility (A*i*) at location *i* (Eq. 2):

2 $$ {A}_i=\sum \limits_{j\in \left\{{d}_{ij}\le {d}_{max}\right\}}w\left({d}_{ij}\right){R}_j $$

where *R*_*j*_ is the bed-to-population ratio of the hospital center *j,* and *d*_*ij*_ is the driving time between the FGC *i* and the hospital center *j*.

All driving times from *i* to *j* were obtained using Google Maps and then computed by SAS version 9.3 [56]. The E2SFCA accessibility score was calculated with the MYSQL program. The definition of the decay function *w()* and time thresholds were previously explained [32]. Briefly, when the travel time to a MCO and to a SSR facility was longer than 41 and 69 min, respectively, that hospital was considered too distant to be accessible. These distance decay parameters were used as cut-off distances to define the catchment areas. The spatial accessibility index *A*_*i*_ obtained with the E2SFCA method is a special form of the physician-to-population ratio, expressed as the number (N) of beds per 10, 000 inhabitants. Higher scores indicate higher accessibility.

#### Measuring health service utilization using the LOS indicator

The LOS described the mean hospital stay duration of each age group (75–84, 85–94 and > 95 years) relative to the whole ≥75-year-old population in that FCG (Eq. 3):

3 $$ {LOS}_i=\sum \limits_{g>75}\frac{Average\ length\ {of\ stay}_{gi}}{P_{gi}} $$

where *gi* represents the three age groups for a given spatial unit i, and *Pgi* the corresponding total population for that age group.

#### Linear regression model and composite accessibility indicator by principal component analysis

A multiple ordinary least squares (OLS) regression model was used to investigate the relationship between LOS ([LOS_MCO]_hospital and [LOS_SSR]_hospital as the dependent variable), the hospital accessibility indicators ([ISA_MCO]_hospital and [ISA_SSR] _hospital), and the accessibility to the three non-hospital practitioners: general practitioners, physiotherapists, and home-visiting nurses ([APL]_non-hospital) (Eq. 4). As our variables were log-normally distributed, they were normalized using the logarithmic Napierian function.

4 $$ \mathit{\ln}\left({LOS}^{\ast}\right)={\beta}_0+{\beta}_1\cdot \mathit{\ln}\left({ISA}^{\ast}\right)+\sum {\beta}_n\cdot \ln \left({APL}_n\right)+\varepsilon; with\kern0.5em \varepsilon \approx iid\left(0;{\sigma}^2\right) $$

where * indicates the [LOS_MCO]_hospital (with the corresponding [ISA_MCO]_hospital as independent variable) and [LOS_SSR]_hospital (with the corresponding [ISA_SSR]_hospital as independent variable), and *n* defines the number of different types of non-hospital healthcare professionals considered in the analysis (*n* = 3: general practitioners, physiotherapists, and home-visiting nurses).

To describe the global accessibility to the three non-hospital services, a composite APL index was built using principal component analysis. In Eq. (5), the three [APL]_non-hospital types are replaced by the [Composite_APL]_non-hospital. This approach offers the advantage of taking into account the correlation between each [APL]_non-hospital value to assess the accessibility to non-hospital services.

5 $$ \mathit{\ln}\left({LOS}^{\ast}\right)={\beta}_0+{\beta}_1.\mathit{\ln}\left({ISA}^{\ast}\right)+{\beta}_2.\mathit{\ln}\left( Composite\  APL\right)+\varepsilon; with\ \varepsilon \approx iid\left(0;{\sigma}^2\right) $$

In summary, Eqs. (1) and (2) were used to estimate the [ISA_MCO]_hospital and [ISA_SSR]_hospital variables. Eq. (3) was used to estimate the [LOS_MCO]_hospital and [LOS_SSR]_hospital variables that are Y variables in the regression model. Eq. (4) is the OLS regression model to investigate the relationship between LOS, inpatient hospital care accessibility and accessibility to the three non-hospital services. Eq. (5) is a variation of Eq. (4) in which the three [APL]_non-hospital types were replaced by the [Composite_APL]_non-hospital variable to take into account their correlation.

## Results

### Descriptive analysis and spatial distribution of the [ISA_MCO]_hospital and [ISA_SSR]_hospital values

In total, there were 240 FGC units in the Nord administrative region. Table 2 summarizes the [ISA_MCO]_hospital and [ISA_SSR]_hospital values. The mean numbers (standard deviation) of beds in MCO and SSR were 19.0 (10.6) and 5.58 (2.23) for 10,000 inhabitants, respectively, highlighting important variations among FGC units. Almost 25% of the population had access to fewer than 10 beds in MCO and 4 beds in SSR.

**Table 2:** Descriptive analysis of the Index of Spatial Accessibility (ISA) for MCO and SSR. Nord administrative region (expressed for 10,000 inhabitants)

	N	Min	Mean (Sd)	Max	25th	Median	75th
MCO	240	0.44	19.0 (10.69)	39.28	10.28	14.32	29.41
SSR	240	0.03	5.58 (2.19)	9.00	4.25	6.33	7.25

*MCO* Medical, Surgical and Obstetrics, *SSR* Postoperative and Rehabilitation Care; *Sd* Standard deviation

**Fig. 1:**
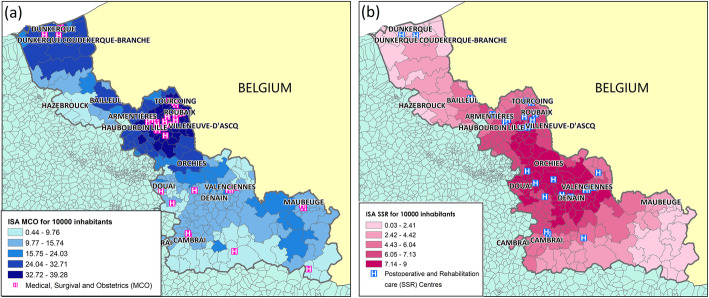
Spatial distribution of the Index of Spatial Accessibility at the French Geographic Code level. **a** Index of Spatial Accessibility for Medical, Surgical and Obstetrics (MCO) and **b** Post-operative and Rehabilitation Care (SSR) centers. For each map, the French neighboring administrative regions are colored in green, whereas the Nord administrative region is represented using a graduated color approach, to highlight the different ISA scores. Maps drawn by Fei GAO

To compare the spatial distribution of the [ISA_MCO]_hospital (a) and [ISA_SSR]_hospital (b) values per 10,000 inhabitants (Fig. 1) within the Nord administrative region, scores were categorized in five classes (from low to high accessibility), using the Jenks Natural Breaks algorithm [57]. The Jenks Natural Breaks algorithm assigns values to a given number of classes with the objective of minimizing the variance within classes, while maximizing the between-class mean values. The [ISA_MCO]_hospital values ([24.04; 32.71] and [32.72; 39.28]) were highest in urban areas close to Dunkerque (the northern part of the region under study), and also in the center, around Lille, Roubaix and Tourcoing. On the other hand, these values were very low towards the south and around Hazebrouck. The [ISA_SSR]_hospital values were highest in the central area ([6.05; 7.14] and [7.14; 9]), and decreased in the north and south. These findings showed that accessibility to hospital services is higher for people in large urban areas, despite the dense population and consequently the higher demand.

### Older adults and [LOS]_non-hospital spatial distribution

**Fig. 2:**
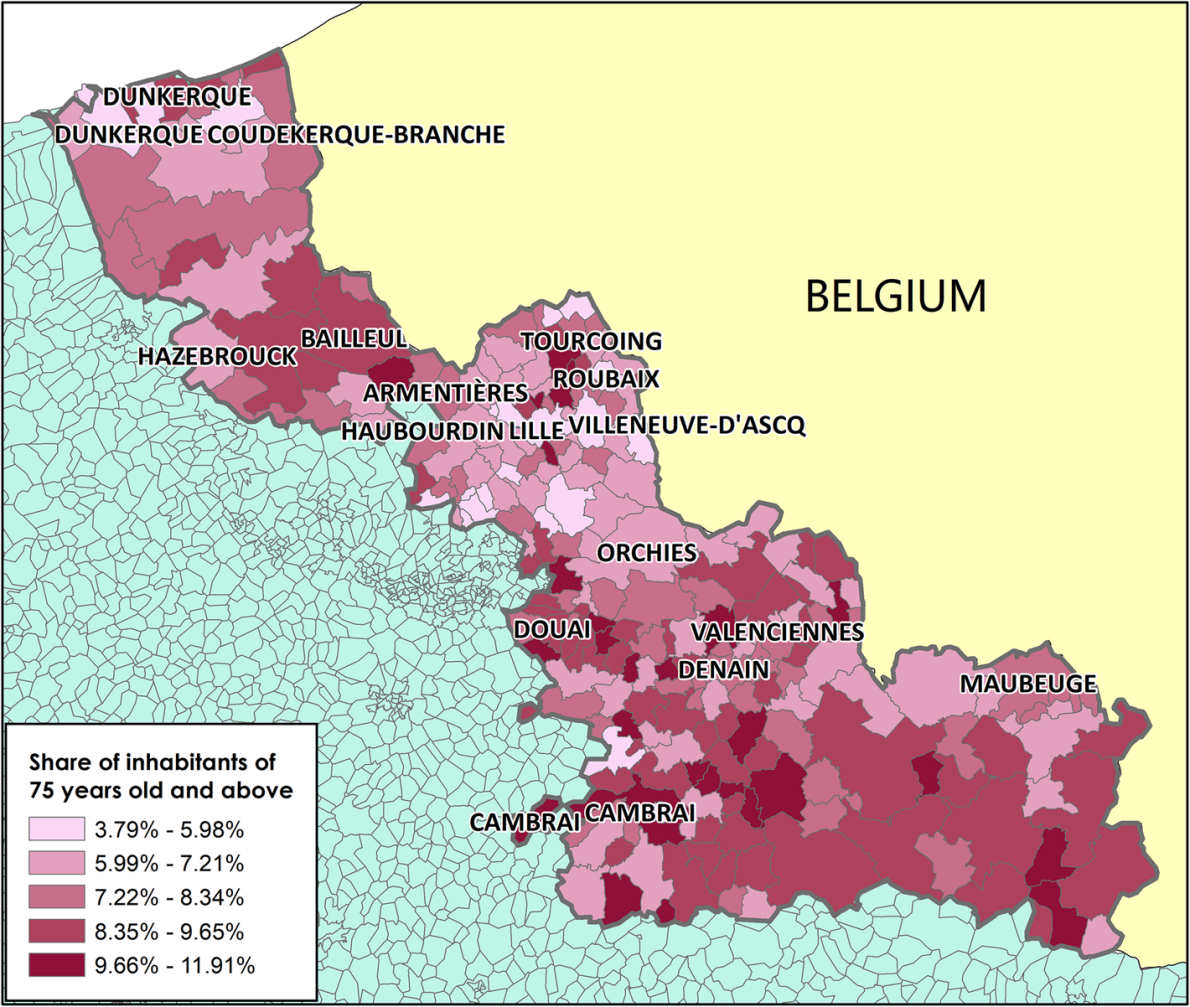
Distribution of the ≥75-year-old population at the French Geographic Code level in the Nord administrative region in 2016. Maps drawn by Fei GAO

The ≥75-year-old population was not homogeneously distributed over the studied territory. Their percentage varied from 3.79% (in the north-west area around Dunkerque, and in the center, particularly the cities of Roubaix, Lille and Villeneuve d’Ascq) to 11.91% (around Bailleul and Hazebrouck and below the Valenciennes-Cambrai line) (Fig. 2).

**Fig. 3:**
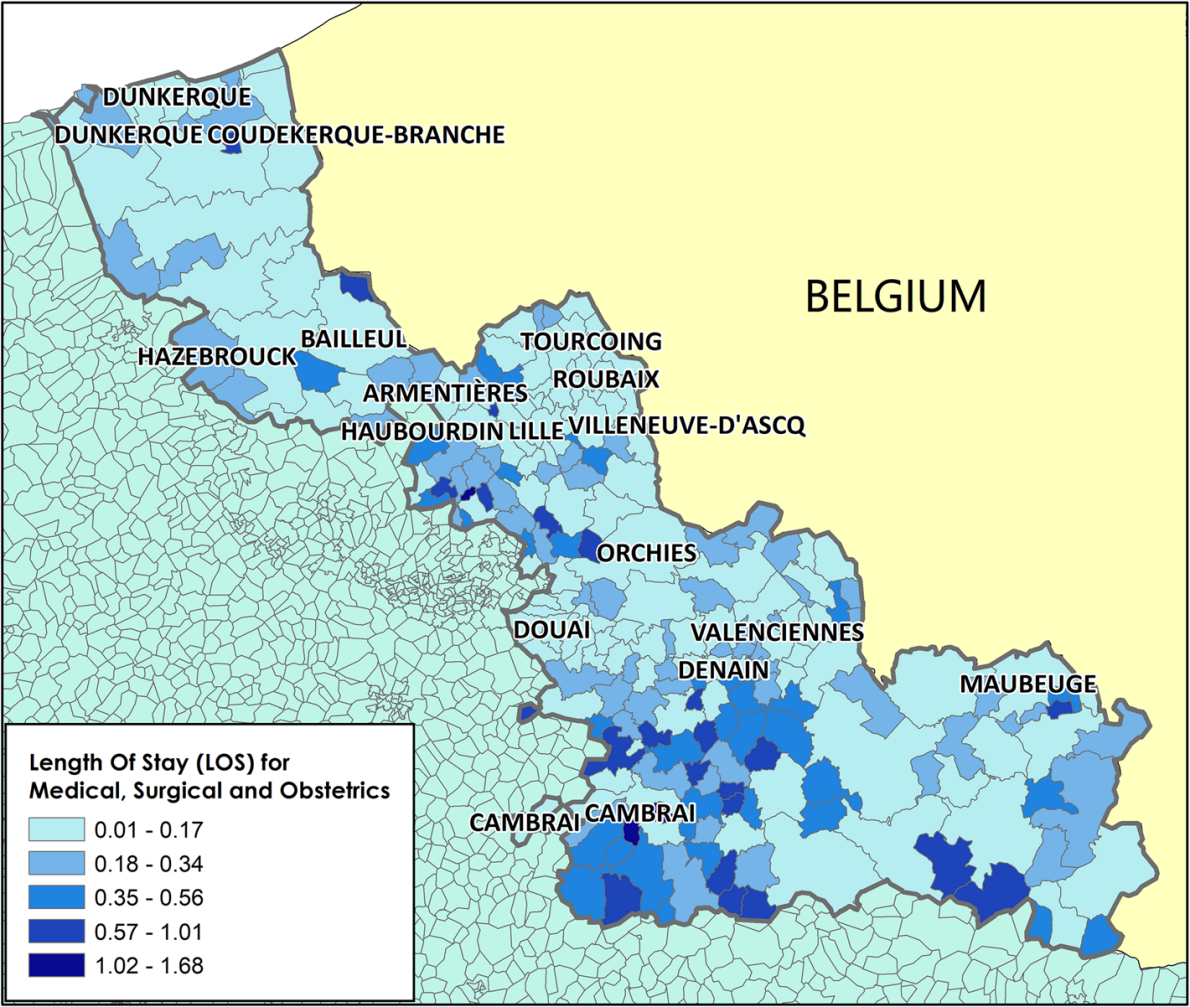
Spatial distribution of length of stay at Medical, Surgical and Obstetrics (MCO) facilities for ≥75-year-old people at the French Geographic Code level (Nord administrative region). Length of stay (LOS) for Medical, Surgical and Obstetrics (MCO) centers. Map drawn by Fei GAO

**Fig. 4:**
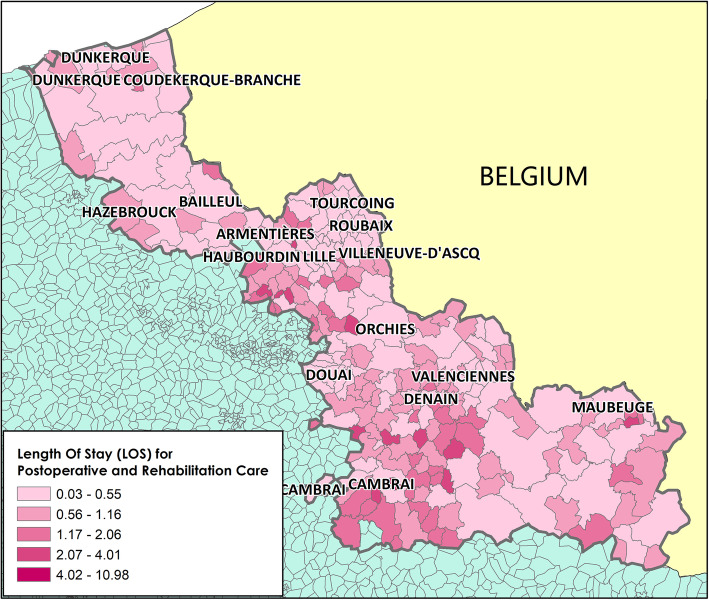
Spatial distribution of length of stay at post-operative and Rehabilitation Care for ≥75-year-old people at the French Geographic Code level (Nord administrative region). Length of stay (LOS) for Post-operative and Rehabilitation Care (SSR) centers. Map drawn by Fei GAO

Analysis of the [LOS_MCO]_hospital and [LOS _SSR]_hospital values for the ≥75-year-old population in the Nord administrative region (Table 3) showed that in 2014, the mean [LOS _MCO]_hospital and [LOS_SSR]_hospital values were 0.26 and 0.85, respectively. The spatial variation was significant, with standard deviations of 0.20 and 0.92 for MCO and SSR, respectively. LOS distribution highlighted a non-homogeneous repartition, suggesting the existence of spatial dependencies in LOS distribution within the studied territory (Figs. 3 and 4). This hypothesis was confirmed by the Moran test results (*p*-value = 0.000 for both [LOS_MCO]_hospital and [LOS _SSR]_hospital scores).

**Table 3:** LOS of ≥75-year-old people in MCO and SSR facilities – Nord administrative region

	N	Min	Mean (Sd)	Max	25th	Median	75th
MCO	240	0.01	0.26 (0.20)	1.68	0.10	0.20	0.37
SSR	240	0.03	0.85 (0.92)	10.98	0.33	0.65	1.08

*MCO* Medical, Surgical and Obstetrics, *SSR* Postoperative and Rehabilitation Care; *Sd* Standard deviation

### Determinants of the [LOS_MCO]_hospital and [LOS _SSR]_hospital values

The OLS regression analysis was used to investigate the determinants of the [LOS_MCO]_hospital and [LOS _SSR]_hospital scores separately at the FGC scale. This analysis (the regression coefficient estimates and the corresponding *p*-values are in Table 4) showed that for the [LOS_MCO]_hospital score, two variables were significant (*p*-value < 0.01): global spatial accessibility to the three types of non-hospital care services ([Composite_APL]_non-hospital) and spatial accessibility to MCO facilities ([ISA_MCO]_hospital). Both variables revealed a strong negative association, suggesting that LOS were shorter for patients living in areas with easier access to non-hospital care services and MCO facilities. The SSR analysis also suggested that better accessibility to non-hospital care services might decrease LOS. However, the spatial accessibility to SSR facilities became non-significant.

**Table 4:** OLS regression analysis of length of stay (LOS) at MCO and SSR facilities

[LOS_MCO]_hospital		
Predictor variables	Coefficient	Std Error
[Composite_APL]_non-hospital	−1.141***	0.201
[ISA_MCO]_hospital	−.283**	0.087
Observations	240	
R^2^	0.134	
VIF	< 1.17	
[LOS_SSR]_hospital		
Predictor variables	Coefficient	Std Error
[Composite_APL]_non-hospital	−1.379***	0.118
[ISA_SSR]_hospital	.045	0.069
Observations	239	
R^2^	0.082	
VIF	< 1.13	

** *p* < 0.01; *** *p* < 0.001; *MCO* Medical, Surgical and Obstetrics, *SSR* Postoperative and Rehabilitation Care, *Std Error* Standard deviation Error, [Composite_APL]_non-hospital: Built from Localized Potential Accessibility to general practitioners, home-visiting nurses and physiotherapists using principal component analysis; [ISA_MCO]_hospital: index of spatial accessibility to SSR facilities; [ISA_SSR]_hospital = index of spatial accessibility to SSR facilities

## Discussion

### Summary of the results

To our knowledge, this is the first study to investigate the relationship between healthcare utilization (LOS) and spatial accessibility to inpatient hospital care facilities ([ISA]_hospital) and three types of non-hospital care services ([APL]_non-hospital, for general practitioners, physiotherapists, and home-visiting nurses). One of its main strengths is the cross-referencing of different data sources that allowed us to address several major public health issues.

First, following a previously developed methodology [32, 54], we estimated a measure of spatial accessibility to hospital care, for MCO and SSR facilities separately, at the FGC scale (approximately equivalent to the municipality scale). All previous French studies that used a similar methodology limited their accessibility measure to non-hospital care services [30–32]. The present study originality lies in extending the measure to hospital spatial accessibility, by taking into account the number of beds in MCO and SSR facilities, the car travel time, and the population distribution. The [ISA_MCO]_hospital and [ISA_SSR]_hospital variables were initially developed at the census block scale, then summarized to the FGC scale to investigate the association with the [LOS]_hospital indicator, because FGC is the smallest spatial level for which hospital data are available. Even at this spatial scale, the [ISA]_hospital score distribution highlighted the unequal accessibility to MCO and SSR facilities within the study area. Analysis of the [ISA]_hospital score spatial distribution revealed that high-accessibility areas were mainly concentrated in the center of the investigated area. This can be partly explained by the large number of MCO and SSR facilities (25 of the 44 MCO and 24 of the 30 SSR centers) in these areas. However, we observed exceptions. Around Valenciennes, where there are three MCO hospitals with more than 400 beds in total, the [ISA]_hospital values were in the lower class. Conversely, the [ISA]_hospital value for Bailleul was quite high, although no MCO facility was located in or close to this city. This finding is in agreement with the fact that the [ISA]_hospital variable provides a summary measure of two important and related components of accessibility: the volume of services available relative to the population size, and the proximity of services available relative to the population location. Therefore, although 400 MCO beds were located close to Valenciennes, the population’s size was too important to obtain a high accessibility score*.*

Analysis of the [LOS_MCO]_hospital and [LOS _SSR]_hospital score distribution in the studied region highlighted a non-homogeneous repartition with higher values close to the border with other French regions, especially in the southern part. From Dunkerque to Bailleul and also around Tourcoing, Roubaix, Lille and Orchies, LOS scores were lower. Then, we examined the association between the healthcare utilization indicator ([LOS]_hospital) and two accessibility scores ([ISA]_hospital and [APL]_non-hospital). Our analysis revealed a significant and negative association between the [LOS]_hospital and [Composite_APL]_non-hospital scores. In other words, better accessibility to these non-hospital services corresponded to shorter hospital stays. One hypothesis is that in areas with better accessibility to the three non-hospital care services, hospital stays are shorter because of the presence of effective outpatient care: ambulatory care and neighborhood healthcare services. For instance, home-visiting nurses and physiotherapists could be an alternative solution to SSR inpatient care. These results further support the hypothesis of complementary interactions between non-hospital and hospital services. These first findings should be complemented by research to determine the impact of primary care accessibility on length of stay.

### Comparison with the international literature

Previous studies have investigated healthcare spatial accessibility and the question of whether healthcare activity could be rebalanced by expanding/strengthening the role of primary care relative to the more expensive hospital (secondary) care. Most works on the use of primary care to reduce specialty/inpatient care were observational studies in which the rates of preventable hospitalizations were correlated with the self-rated access level [33] or with the distance [58] to primary care services [59]. Few studies quantified both hospital and non-hospital care spatial accessibility with the E2SFCA method, and investigated their association with the length of hospital stay. The present study fills this gap by integrating three factors: spatial accessibility (1) to inpatient hospital care facilities and (2) to three types of non-hospital care (general practitioners, physiotherapists, and home-visiting nurses), and (3) LOS. As few studies have considered all three with a similar study design, comparison with the international literature was difficult. However, some articles investigated one or two of these aspects. First, although this is the first French study measuring hospital spatial accessibility using the E2SFCA method, other countries, for instance China [60] and Japan [61], already developed hospital accessibility scores following a similar approach. Second, other studies estimated the LOS to assess how primary care could contribute to reduce the demand of secondary care. In France, a study used the LOS for public-sector psychiatric facilities to investigate whether the development of alternatives to full-time hospitalization (such as ambulatory care, part-time hospitalization, and full-time outpatient care) may reduce the LOS [21]. They found a significant negative association, and concluded that their study provided the first nation-wide evidence of the benefits of alternatives to full-time hospitalization in psychiatry. Similarly, our study show that non-hospital care services may reduce the length of stay in MCO and SSR facilities. Together, these findings suggest that in some cases, non-hospital care services may constitute an alternative to hospitalization. Our results were obtained by modeling the association between healthcare utilization and accessibility to two types of healthcare services. These preliminary quantitative results should be completed with data on other healthcare outcomes frequently associated with the quality of care, such as unplanned readmission and mortality, as well as other aspects of accessibility (e.g. multiple consensual indicators of spatial/non-spatial healthcare access). Additional studies using sophisticated modeling methods should also be developed. The goal is to build a consolidated approach to facilitate the spatial organization of non-hospital medical services in the territory with the aim of complementing hospital services and increasing healthcare efficiency.

### Limitations

As we used aggregated data at the FGC scale to assess associations between spatial accessibility to hospital and to three types of non-hospital care services and healthcare utilization, our findings may be subject to an ecological bias [32]. In addition, as previously explained, while the [ISA]_hospital index was estimated at the census block scale, the two other indicators ([LOS]_hospital and [APL]_non-hospital) were only available at the FGC scale, a cruder spatial scale. Thus, we could not take into account the spatial accessibility heterogeneity at the census block scale. For future research, we want to construct a LOS indicator at a finer scale using disaggregation techniques that take into account the population density. Moreover, to compare potential and realized access we used metrics to describe spatial accessibility to inpatient and primary healthcare services/facilities and the use of hospital services (LOS). Unfortunately, an indicator of primary care service utilization is still lacking in France.

In our analysis, we did not use statistical techniques that consider spatial autocorrelation. However, at the FGC scale, the Moran’s indicator revealed the presence of spatial autocorrelation for both MCO and SSR LOS. To precisely investigate the association of healthcare accessibility and utilization, the next step could be to include the specific topological, geometric and geographic characteristics of the study area using spatial statistical models, such as the simultaneous autoregressive, geographically weighted regression and Bayesian hierarchical models. Previous studies [62–66] demonstrated the robust properties of these models that can improve the methodology used to assess associations between healthcare spatial accessibility and utilization. For instance, Nicholas et al. used Bayesian spatial models and location analysis methods to evaluate healthcare facility access [67].

## Conclusion

Our study brings two main contributions. From a methodological point of view, this is the first study to measure spatial accessibility to MCO and SSR facilities in France using the E2SFCA method and to investigate the relationship between spatial accessibility to inpatient hospital care facilities and to non-hospital care services. Regarding the practical aspect, it provides a basic understanding of the inpatient care status within the studied area by showing the accessibility score variation across the territory, and by highlighting some areas with poor accessibility. This type of information is important to guide policy makers and local managers. Moreover, this study explored the interactions between healthcare service access and utilization. Our findings support the hypothesis of complementary effects between non-hospital and hospital services. Based on our results, policy makers and local managers could identify areas where additional beds or healthcare professionals should be allocated in priority.

These results need now to be confirmed by additional studies in other geographical areas. It is also crucial to design new research approaches to understand the underlying mechanisms and processes that explain the interaction between inpatient hospital and non-hospital care services with the ultimate objective of better organizing and allocating medical resources. This research should help to make decisions about deploying additional beds and identifying the best locations for non-hospital care services, and also to improve access, to ensure the best coordination and to contribute to the sustainability of inpatient care and outpatient services, in order to better meet the population’s health needs.

## Data Availability

All data generated or analyzed during this study are included in this published article. If readers need supplementary information or request the data, they can contact me (fei.gao@ehesp.fr).

## Abbreviations

APL: Localized Potential Accessibility
E2SFCA: Enhanced two-Step Floating Catchment Area
FINESS: National File on Health and Social Institutions
FGC: French Geographic Code unit
ISA: Spatial Accessibility Index
MCO: Medical, Surgical and Obstetrics
SAE: Annual Statistical Survey of Healthcare Facilities
SSR: Postoperative and Rehabilitation Care
LOS: Length Of Stay
OLS: Ordinary Least Squares
